# Supplementary material for: Fabrication of Salvinia-inspired surfaces for hydrodynamic drag reduction by capillary-force-induced clustering
Source: Nat Commun. 2022 Sep 2;13:5181. doi: 10.1038/s41467-022-32919-4 (PMC9440115; doi:10.1038/s41467-022-32919-4)
Supplement: Supplementary file 1 — Supplementary Information [file 41467_2022_32919_MOESM1_ESM.pdf]

Supplementary Information

Fabrication of *Salvinia*-inspired surfaces for hydrodynamic drag reduction by capillary-force-induced clustering

*Minsu Kim[1], Seunghoon Yoo[1], Hoon Eui Jeong[2] and Moon Kyu Kwak[1]**

[1]Department of Mechanical Engineering, Kyungpook National University, Daegu 41566, Republic of Korea
Prof. H. E. Jeong
[2]Department of Mechanical Engineering, Ulsan National Institute of Science and Technology (UNIST), Ulsan 44919, Republic of Korea
*E-mail: mkkwak@knu.ac.kr

Supplementary Figures 1-7
Supplementary Table 1
Supplementary References

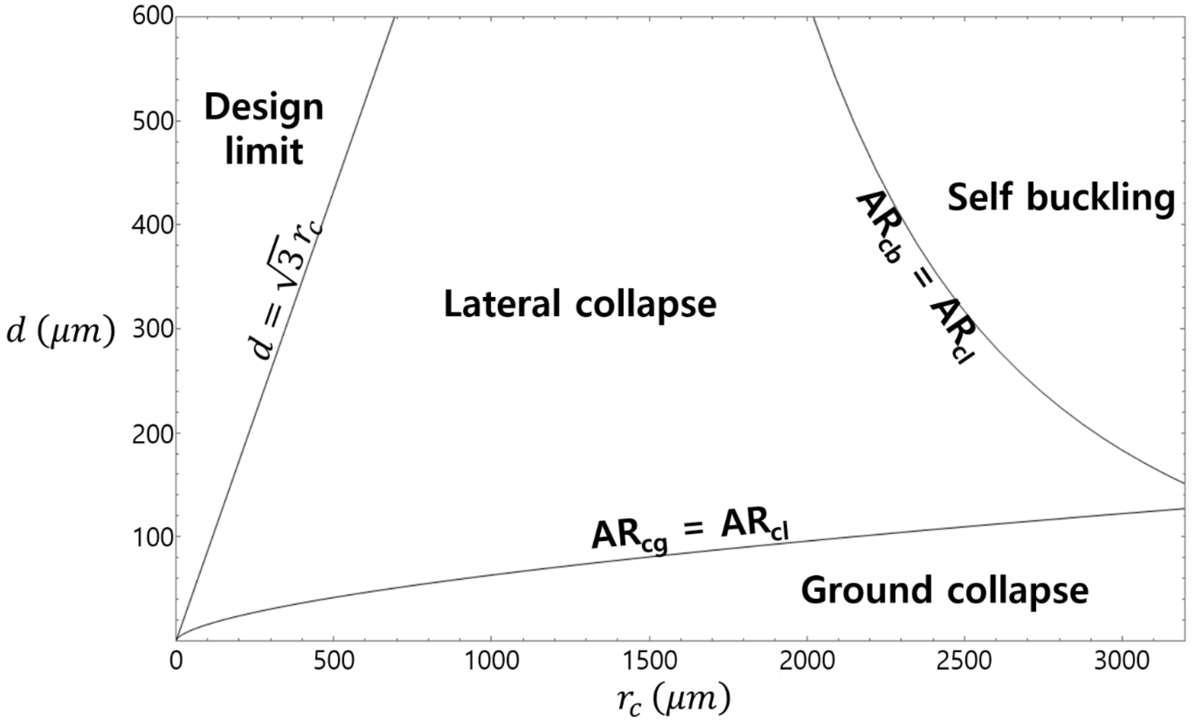

Supplementary Fig. 1.
Design limit and critical condition plots for each collapse type according to the center-to-center radius and pillar diameter.

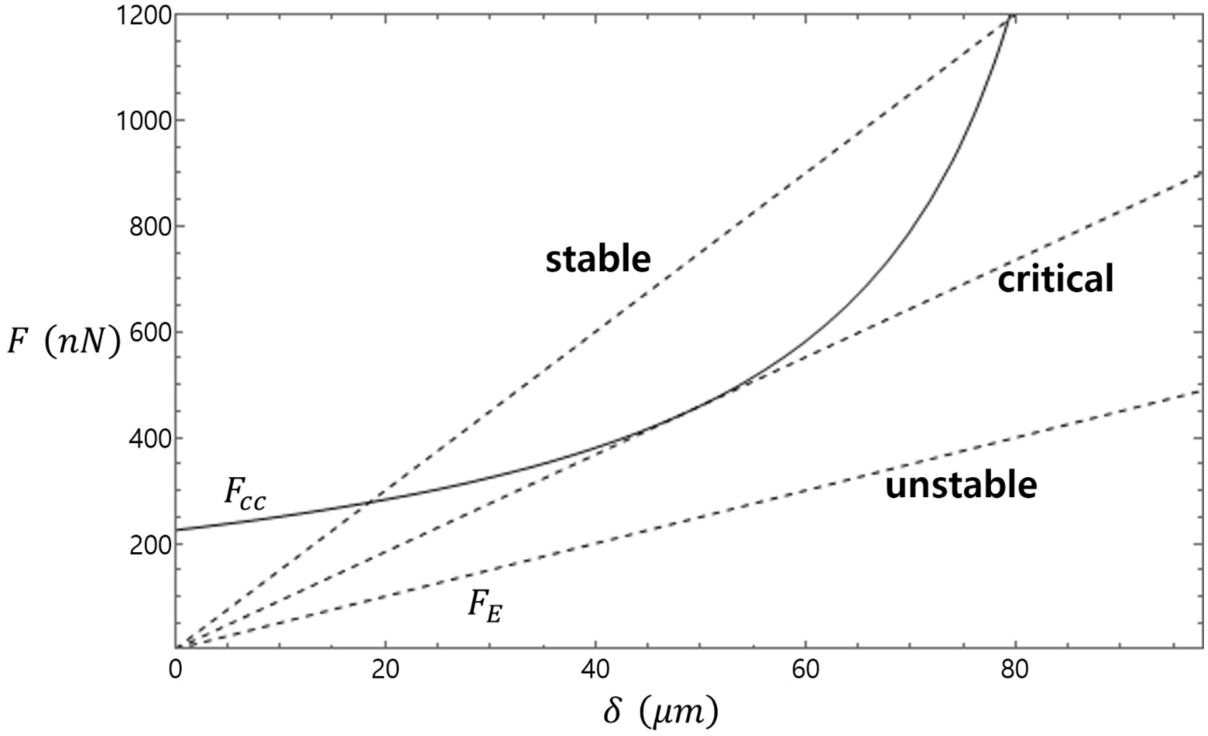

Supplementary Fig. 2.
Typical cases of capillary interaction force and elastic restoring force as a function of pillar deflection.

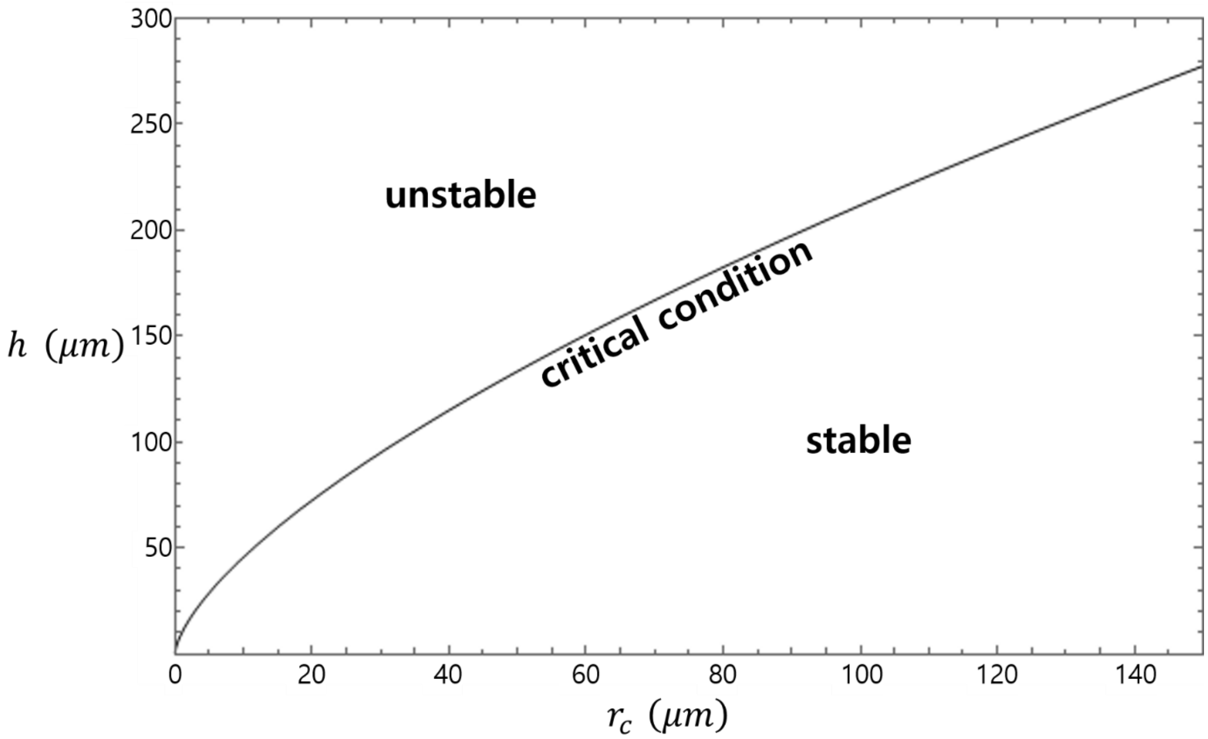

Supplementary Fig. 3.
The critical condition for capillary-force-induced clustering.

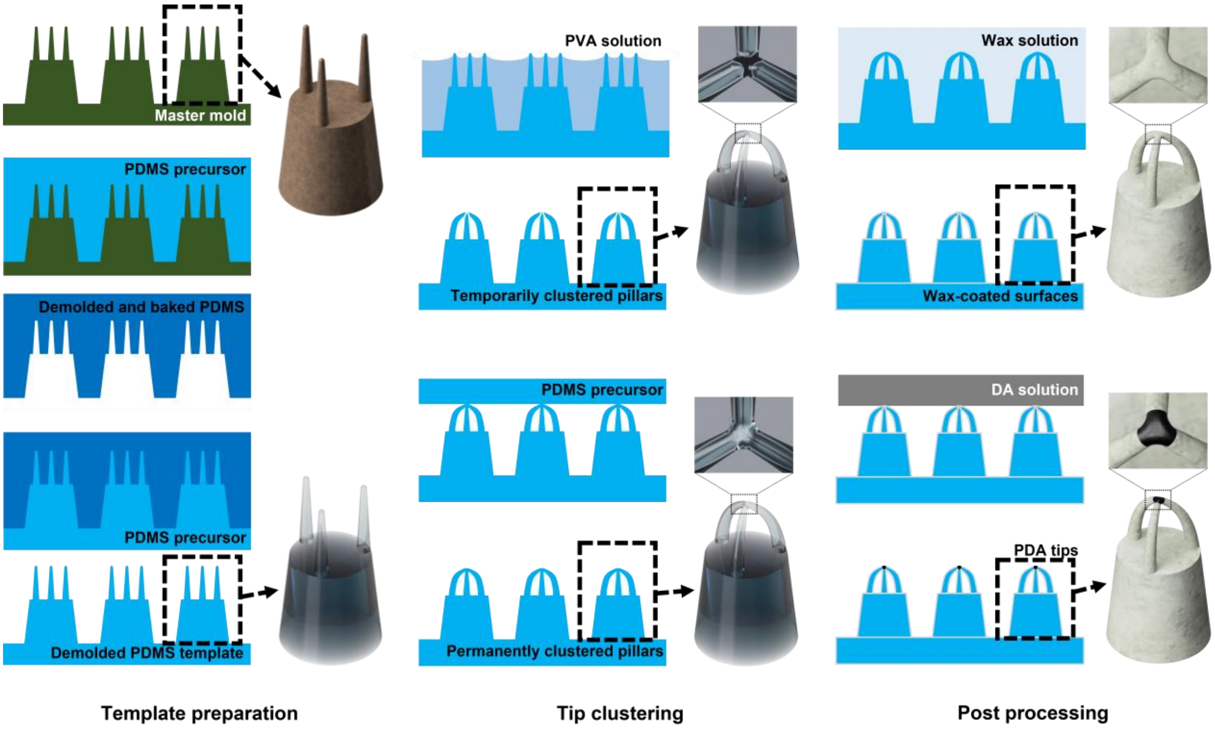

Supplementary Fig. 4.
Schematic illustration of every phase of the fabrication processes.

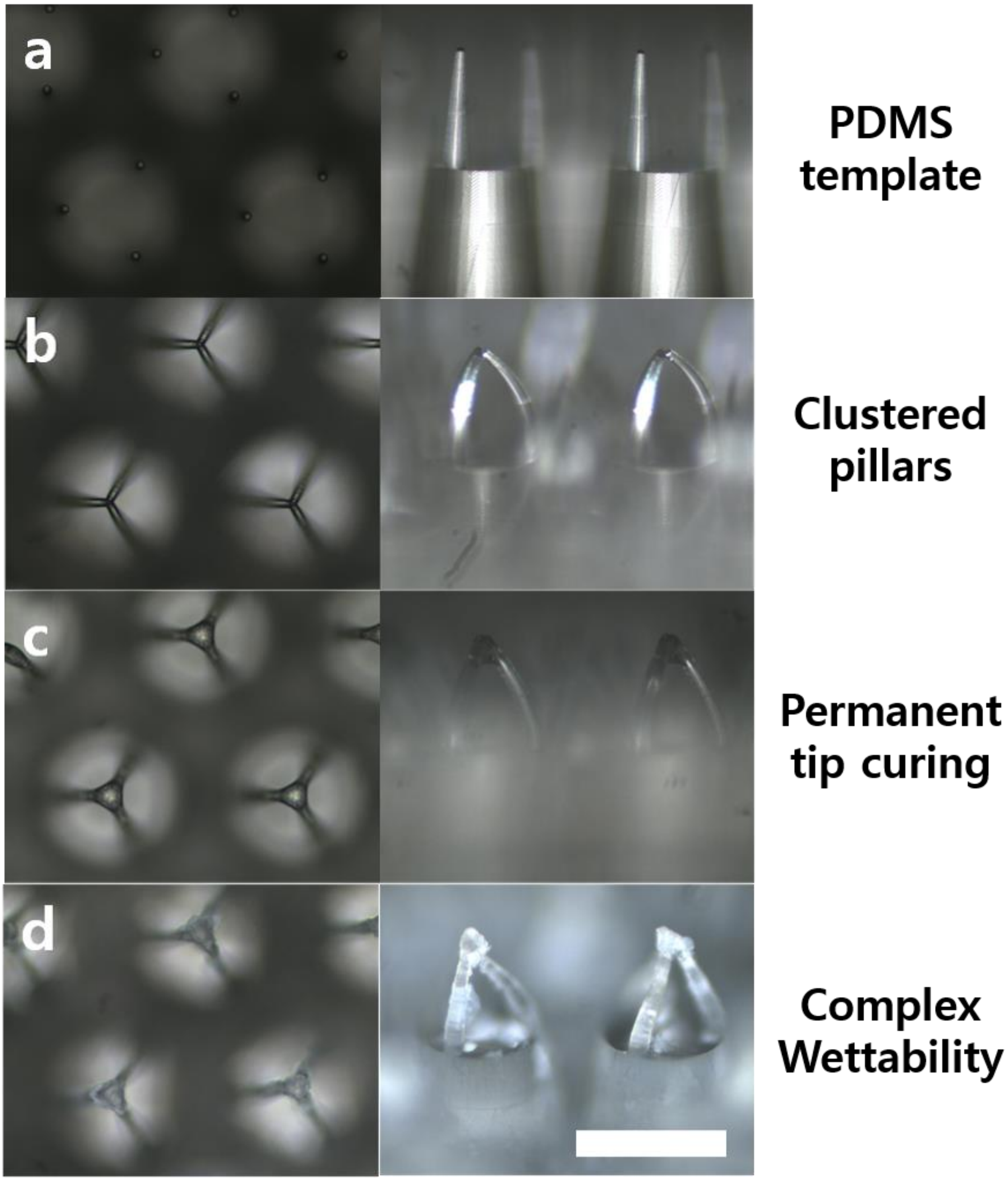

Supplementary Fig. 5.
Microscopic images of PDMS hierarchical pillar array at each fabrication step. Scale bar represents 300 μm.

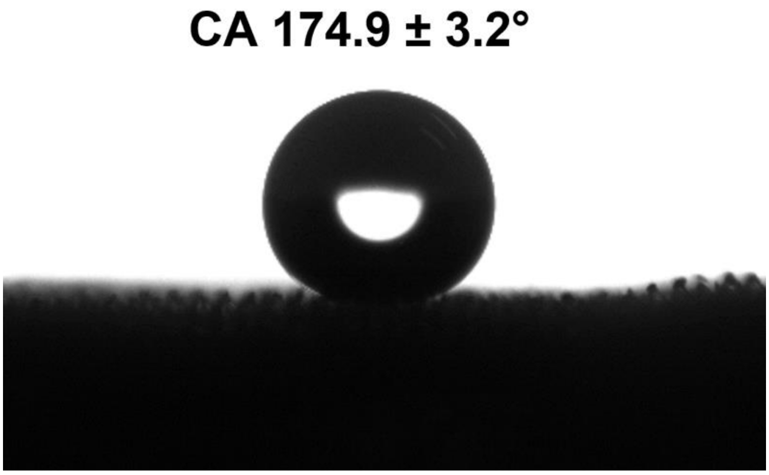

Supplementary Fig. 6.
Water droplet on the fabricated *Salvinia*-inspired surfaces showing superhydrophobicity.

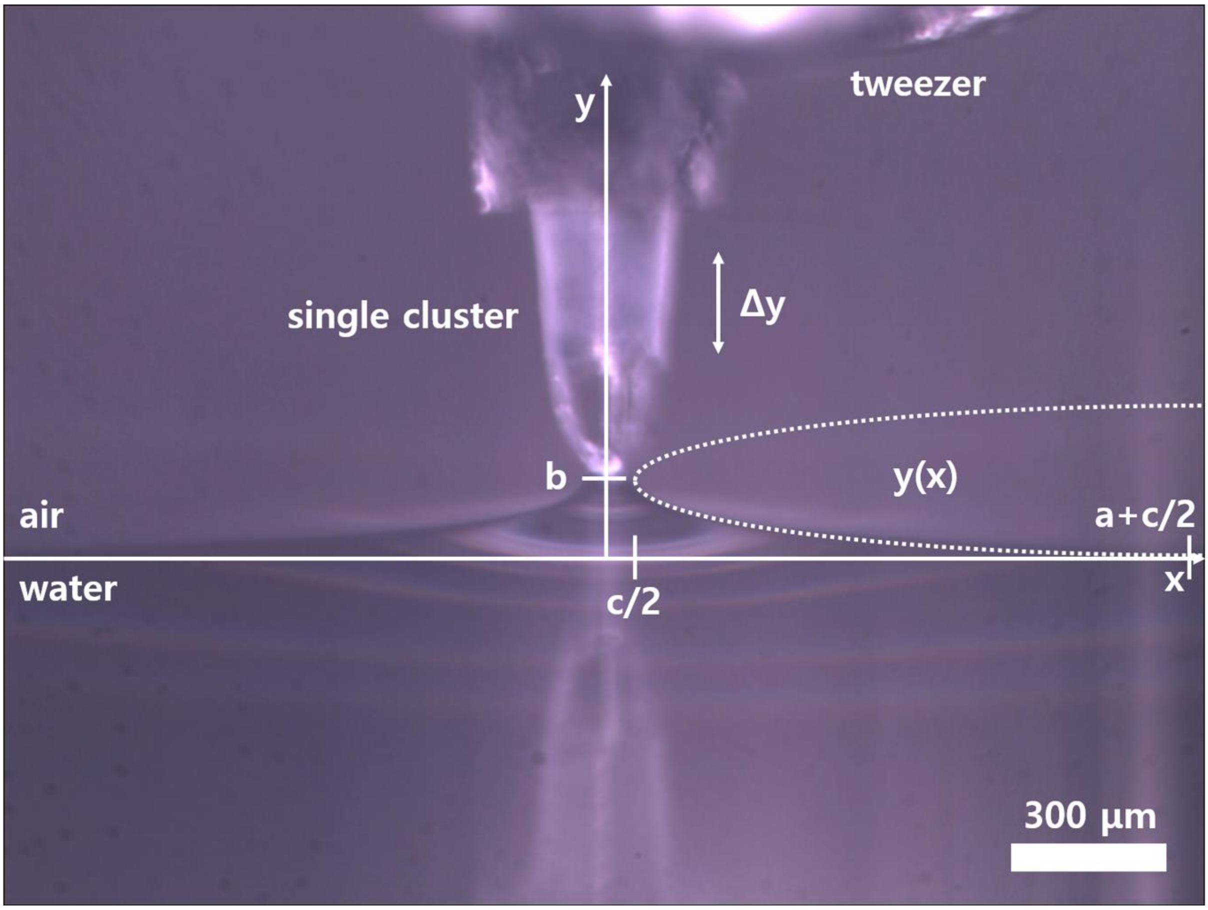

Supplementary Fig. 7.
Microscopic image of a single clustered structure pulling the water meniscus just before the snap-off. Variables for the elliptical assumption are depicted to calculate the meniscus pulling force of the single clustered pillar.

The meniscus pull-off force of a single clustered structure can also be calculated by $C\sigma \cos\theta$, where C is the circumference of contact, σ is the surface tension of the liquid, and θ is the local contact angle[1–3]. Although it is difficult to determine the exact local contact angle θ from the image, with rough approximate measurements the pull-off force can be calculated as

in a range of 18.1 ~ 20.0 μN, which is in a similar order with the value calculated from the elliptic approximation method following Gandyra et al. (Beilstein J. Nanotechnol., 2015)[4].

Supplementary Table 1. Physical properties of PDMS(Sylgard 184)

Physical property	Value	Unit
Elastic modulus	1.09	Mpa
Poisson's ratio	0.499	-
Surface energy	19.8	mJ/m^2
Density	965	Kg/m^3

Supplemetary References

1. Dynes, P. J. & Kaelble, D. H. Surface energy analysis of carbon fibers and films. *J. Adhesion* **6**, 195–206 (1974).
2. Schultz, J., Cazeneuve, C., Shanahan, M. E. R. & Donnet, J. B. Fibre surface energy characterization. *J. Adhesion* **12**, 221–231 (1981).
3. Alimov, M. M. & Kornev, K. G. Meniscus on a shaped fibre: singularities and hodograph formulation. *Proc. R. Soc. A* **470**, 20140113 (2014).
4. Gandyra, D. *et al*. The capillary adhesion technique: a versatile method for determining the liquid adhesion force and sample stiffness. *Beilstein. J. Nanotechnol.* **6**, 11-18 (2015).
